# Self-Management of Subclinical Common Mental Health Disorders (Anxiety, Depression and Sleep Disorders) Using Wearable Devices

**DOI:** 10.3390/ijerph20032636

**Published:** 2023-02-01

**Authors:** Tony Robinson, Joan Condell, Elaine Ramsey, Gerard Leavey

**Affiliations:** 1 School of Computing, Engineering, and Intelligent Systems, Ulster University, Magee Campus, Derry/Londonderry BT48 7JL, UK; 2 Department of Global Business and Enterprise, Ulster University, Magee Campus, Derry/Londonderry BT48 7JL, UK; 3 The Bamford Centre for Mental Health and Wellbeing, School of Psychology, Ulster University, Coleraine Campus, Cromore Rd., Coleraine BT52 1SA, UK

**Keywords:** anxiety, depression, wearables, e-mental health

## Abstract

Rationale: Common mental health disorders (CMD) (anxiety, depression, and sleep disorders) are among the leading causes of disease burden globally. The economic burden associated with such disorders is estimated at $2.4 trillion as of 2010 and is expected to reach $16 trillion by 2030. The UK has observed a 21-fold increase in the economic burden associated with CMD over the past decade. The recent COVID-19 pandemic was a catalyst for adopting technologies for mental health support and services, thereby increasing the reception of personal health data and wearables. Wearables hold considerable promise to empower users concerning the management of subclinical common mental health disorders. However, there are significant challenges to adopting wearables as a tool for the self-management of the symptoms of common mental health disorders. Aims: This review aims to evaluate the potential utility of wearables for the self-management of sub-clinical anxiety and depressive mental health disorders. Furthermore, we seek to understand the potential of wearables to reduce the burden on the healthcare system. Methodology: a systematic review of research papers was conducted, focusing on wearable devices for the self-management of CMD released between 2018–2022, focusing primarily on mental health management using technology. Results: We screened 445 papers and analysed the reports from 12 wearable devices concerning their device type, year, biometrics used, and machine learning algorithm deployed. Electrodermal activity (EDA/GSR/SC/Skin Temperature), physical activity, and heart rate (HR) are the most common biometrics with nine, six and six reference counts, respectively. Additionally, while smartwatches have greater penetration and integration within the marketplace, fitness trackers have the most significant public value benefit of £513.9 M, likely due to greater retention.

## 1. Introduction

Mental health disorders are the leading causes of disease burden globally [1]. Additionally, 50% of mental disorders develop by age 14 and 75% by 24 [2]. The global prevalence of major depressive disorder in 2020 before the COVID-19 pandemic was 2470.5 cases per 100,000, equivalent to 193 million people [1]. Correspondingly, it is estimated that anxiety disorders in 2020 before the COVID-19 adjustment were 3824.9 cases per 100,000, or 298 million people [1]. Thus, as of 2020, common mental health disorders affect 6.3% of the global population.

The burden associated with anxiety disorders has risen from approximately 76.2 million to 374 million people, or 4802.4 cases per 100,000 in 2020 [1]. Additionally, the burden associated with depressive disorders has risen by approximately 53.2 million to 246 million people, or 3152.9 cases per 100,000 during the same period [1]. Therefore, following adjustment for COVID-19, common mental health disorders affect 8% of the global population, representing a 1.7% increase in the same period. Fusar-Poli et al [2] have suggested that the worldwide cost of mental health is estimated to reach $16.3 trillion by 2030, exceeding that of cardiovascular disease, chronic respiratory disease, cancer, and diabetes. Within the UK specifically, mental health accounted for 7% of all ill health, amounting to £117.9 billion as of 2019, a 21-fold increase since 2009 [3,4].

Few individuals with a mental health disorder receive prompt treatment, with UK mental health targets consistently remaining unmet [5]. For instance, more than one-third of child and adolescent mental health services (CAMHS) and one-fifth of adult mental health services (AMHS) patients wait longer than the 18-week target for treatment [6]. In addition, the COVID-19 pandemic added pressure to already stretched mental health services [7,8].

Globally, pre-Covid, 50–60% of adults with mental disorders may not have received interventions from mental health services; those typically wait for a decade or more [5]. Early intervention is economically attractive, especially at younger ages [3,5,9]. While international funding is available for mental health projects, from 2006–2016, only $409.1 million of $2.04 billion was awarded to projects focused explicitly on mental health [10].

### Health Technology

There is promising evidence that mental health technologies could efficiently extend and enhance mental health services. Technologies encompassing blended therapies, such as internet-delivered CBT, have been demonstrated as a cost-effective and comparable alternative to in-person treatment [11]. Traditionally, mental health technologies take 16 years for acceptance within professional practice [12]. However, the COVID-19 pandemic has acted as a catalyst, causing the almost immediate integration of mental health technologies within practice and giving rise to self-guided interventions such as self-help apps [12]. Such technologies supporting the transition to blended therapies have the potential to offer rapid and cost-effective interventions, monitoring, and education [11]. Despite this paradigm shift and the potential that mental health technologies offer, low engagement, inadequate clinical evidence, and high attrition rates remain challenges that technologies must overcome [12,13,14].

## 2. Background

This section provides critical background information, including the global prevalence of anxiety and depression, the economic burden, and risk factors for mental health. The following section details the current paradigm towards mental health and its shifting focus towards innovative technology-assisted preventative mental health strategies. Specifically, the role of wearables within this new paradigm will be discussed, and the aim and objectives of this review will be introduced.

### 2.1. Defining Common Mental Health Disorders

**Anxiety:** Contextually, appropriate anxiety is a normal response. Anxiety within the context of mental health refers to systematic feelings of anxiety that are both out of context and proportion. According to the world health organisation, international classification of diseases (IDC-11 2022), anxiety or fear related disorders are a group of mental health disorders characterised by feelings of anxiety and fear. Anxiety disorders include generalised anxiety disorder (GAD), panic disorder, phobias, social anxiety disorder, and separation anxiety [15].

**Depression:** Low mood can be a normal emotional state of everyday life. However, depression refers to systematic and recurring feelings of low mood that are both out of context and proportion [16]. According to the world health organisation [15], mood disorders such as depression refer to major depressive disorder (MDD), bipolar depression, dysthymia, seasonal affective disorder, and postnatal depression.

**Sleep disorders:** An umbrella term applied to several sleep-specific disorders [17]. According to the world health organisation [17], sleep disorders include insomnia, sleep apnoea, narcolepsy, restless leg syndrome and random eye movement (REM) sleep behaviour disorder.

**Common mental health disorders:** Common mental health disorders, according to the world health organisation [16], include the range of anxiety, depression and sleep disorders listed above. Anxiety, depression, and sleep disorders are frequently associated and are often co-occurring, adding to their complexity and associated risk [7].

### 2.2. The Promise of Telehealth and the Integration of Health Self-Service

Symptoms of subclinical mental health impede daily function and are experienced by a significant proportion of the population [11,18]. Furthermore, while subclinical symptoms are unrecognised by psychiatric services, they prompt help-seeking behaviours of those affected [11,18]. Early intervention and promotion of better mental health could potentially reduce the growing personal, social, and economic burden of common mental health disorders [11]. Unfortunately, the prevalence of anxiety-related disorders across populations exceeds the service capacity of mental health services, specifically their ability to conduct timely face-to-face therapy sessions with those affected [11,19]. However, there is encouraging evidence to support the implementation of digital, telehealth and face-to-face services tailored to individual needs, thereby improving access to mental health services while also promoting the use of self-management tools, thus potentially reducing burden on such services [1,11,20].

Individuals with “long COVID” may develop depressive and anxiety disorders, adding to the health burden [1,21]. Digital therapies can scale according to demand, extending the reach of mental health services so long as such therapies are comparable to the efficacy of traditional treatments. Firth et al [11] argued that substantial clinical evidence demonstrates the efficiency of internet-delivered cognitive behavioural therapy (CBT) in treating anxiety. Furthermore, 65% of the global population have access to a mobile phone, increasing by 61% over the past decade; thus, smartphone apps for anxiety are a popular and ubiquitous method of intervention delivery, despite their evidence drastically lagging the extensive marketing and commercialisation efforts driving their development [11]. 

Irrespective of the lack of supporting evidence regarding specific implementation from developers, the greater affordability and usability of smartphones and tablet devices have presented new opportunities for the assessment and treatment of psychiatric disorders [11,22]. The recent COVID-19 pandemic acted as a catalyst, causing the almost immediate integration of mental health technologies within clinical practice and giving rise to self-guided interventions such as self-help apps [12]. Wearable device, such as smartwatches, fitness trackers and headbands while traditionally adopted by the health conscious as a means to quantify their progress may extend the efficiency of digital therapies while permitting people access to personal analytics and supporting self-management of conditions [23]. Furthermore, Piwek et al [23] suggest that wearable devices have the potential to provide detailed and cost-effective longitudinal data without the need to involve more sophisticated, uncomfortable, and expensive alternatives. Finally, as individuals seek greater autonomy for their treatment options, wearable data may become a common feature of primary care visits [23].

There is increasing evidence for the use of digital therapies within the clinical setting and the efficacy of smartphone-based digital treatments within community subgroups for well-being [11,12,22]. However, there is scant evidence, particularly from manufacturers, to support the efficacy of wearables for the self-management of common mental health disorders and how these devices could potentially act as innovative tools to scale mental health services provision [23].

## 3. Review Aims and Objectives

**Aims:** This review aims to evaluate the potential utility of wearables for the self-management of sub-clinical common mental health disorders. Furthermore, we seek to understand the potential of wearables to reduce the burden on the healthcare system.

Objectives:

1. Analyse the dominant wearable biometrics currently used in wearable devices as described within the current literature.
2. Analyse the central purpose of wearable devices as described within the current literature.
3. Understand the major machine learning algorithms deployed within these devices.
4. Analyse the potential cost-benefit wearable devices have.

## 4. Methodology

This review follows the Preferred Reporting Items for Systematic Reviews and Meta-Analyses (PRISMA) to explore the aims and objectives above [24]. The PRISMA checklist is provided in Supplementary Materials. The following section details information sources, inclusion and exclusion criteria, and data collection and analysis, including the PRISMA flowchart.

### 4.1. Information Sources

The following databases were used to identify studies for this review: Google scholar, Web of Science, Scopus, PubMed, Cochrane Library, and PsycINFO. Table 1 below shows the initial search terms and their returned results across the databases.

### 4.2. Selection Process

To ensure that current studies are captured within this review a timeframe of 5 years was selected. Similarly, to ensure that reported technologies have generalizable results, a minimum number of participants was taken as n=20.

The following inclusion and exclusion criteria were applied to return results:

Inclusion criteria:

1. Included results within the date range 2018–2022. A five-year search window was chosen due to the exponential growth in wearable technology, the speed of technological development, and to capture the influence of COVID-19 in the results.
2. Included results were common mental disorders (anxiety or depressive disorder, sleeping disorder) as they were the primary focus.
3. Results describe or evaluate e-mental health or wearable technology.

Exclusion criteria:

1. Returned results outside of 2018–2022.
2. Exclude articles with a low number of participants n<20.
3. Exclude articles focused on professional practice and the well-being of healthcare workers within the clinical setting or directly related to occupational stress.
4. Exclude articles focused on professional performance enhancement within sport.
5. Language exclusions: only include English language results.
6. Excluded results did not focus on common mental health conditions (anxiety or depression) as the primary focal point i.e., excluded those that considered mental health a secondary factor to patient care.
7. Excluded studies focused on the clinical outcomes of sensing as part of treatment.
8. Excluded results related to the improvement of psychiatric education practice.

### 4.3. Data Collection and Analysis

**Figure 1:**
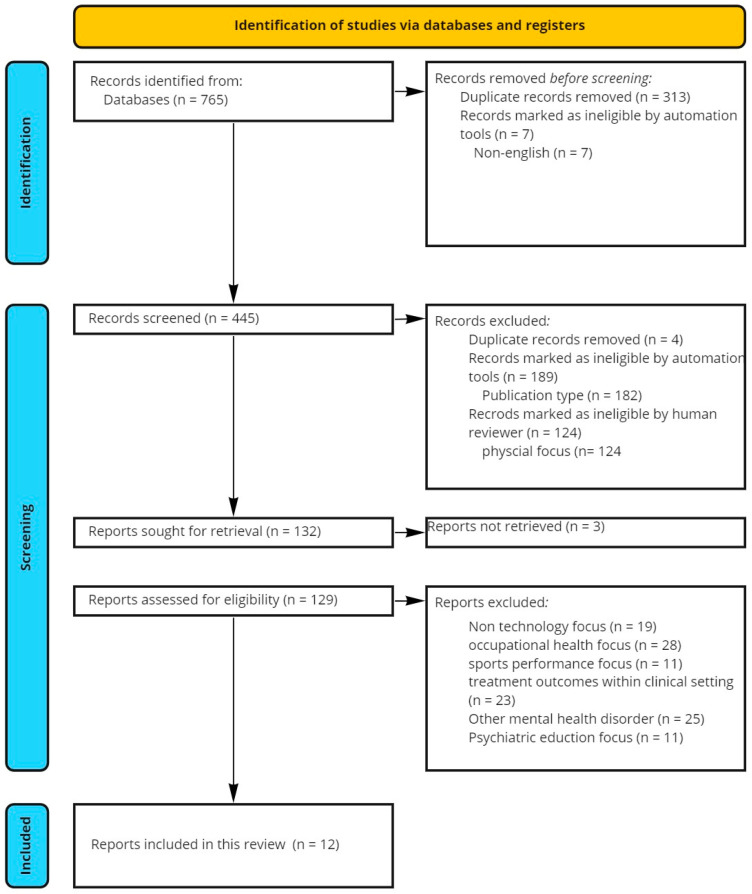
PRISMA flowchart detailing identification, screening, and inclusion of records for this review.

Figure 1 below, illustrates the data identification $\left(n=765\right)$, screening $\left(n=445\right)$ and inclusion $\left(n=12\right)$ process flow as per the PRISMA framework for systematic reviews.

Figure 1 PRISMA flowchart detailing identification, screening, and inclusion of records for this review. The leftmost panels describe the flow between identification, screening, retrieval, eligibility assessment and inclusion. The rightmost panels provide details on each step, including where automation tools have been used and to what effect.

Once the selection of the papers corresponding to the primary studies was concluded, the data contained in their texts were extracted and inserted into structured tables for analysis. The collected data included information about commercial wearables and sensors for remote health monitoring. No randomised controlled selection was performed, as the number of wearables found was small $\left(n=12\right)$. Instead, a single researcher performed the data extraction using manual thematic content analysis on the selected studies. The themes of interest for the data collected on the wearable devices were brand, date released, country of origin, main interface, secondary interface, targeted disorder, data type/biometric used, wearable technology device name and wearable device type model.

The extracted data was formatted and analysed within three categories (1) study characteristics, (2) wearable device biometrics, (3) economic cost–benefit. For each category, the data distribution was analysed with Shapiro–Wilk testing, including fundamental statistical analysis (mean, median, mode, and standard deviation). Pearson’s correlation coefficient was calculated when two or more variables demonstrated a relationship within the included source.

## 5. Results

As per the methodology previously described, 12 studies were identified and included in this analysis. The primary data of interest relates to the wearable device model, wearable device type, machine learning model(s), biometric signals, self-reporting scales, target group, and target disorder. Additionally, passive sensing, related or included interventions and the device’s intended purpose as described within the study were also of interest. The results within this section are organised by (1) study characteristics, exploring the demographic, target disorder and intended purpose metrics. (2) technology and biometric characteristics, and finally, (3) public value benefit analysis using the Greater Manchester cost-benefit analysis (CBA) model [25].

### 5.1. Study Characteristics

**Figure 2:**
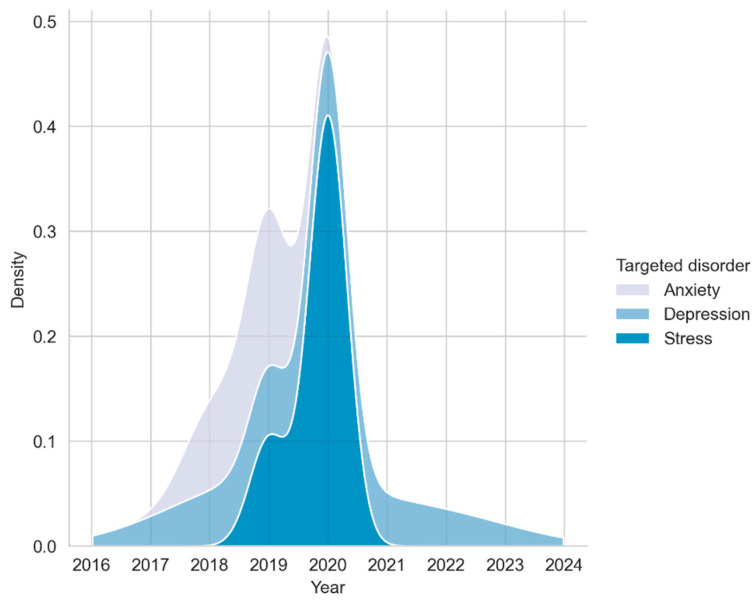
Kernel density estimation (KDE) of targeted disorder by date released.

Figure 2 indicates the kernel density estimation (KDE) of targeted disorder by date released. The data indicates a primary clustering around 2020 with many devices targeting ‘stress’ or ‘depression’.

As illustrated in Table 2, of the studies included in this review (n=12), the majority of publications are clustered around the year 2020, demonstrating a normal distribution per the Shapiro–Wilk test (W=0.874,  P=0.074) with a sample range from 20–5895, mean $\left(M\right)=2019.5,$ standard deviation $\left(SD\right)=1.09$. Included are a total of 7287 participants across all 12 studies (M=607.25,  SD=0.18,  Min=20,  Max=5895). Of the target groups, most studies focused on employees (n=4), followed by university students $\left(n=3\right)$. The least popular group was young and old adults, with n=1 for both. The most common targeted disorder was general stress $\left(n=5\right)$, followed by depression $\left(n=4\right)$. Anxiety is only featured when combined with other targets such as stress $\left(n=2\right)$ or stress and depression $\left(n=1\right)$. Similarly, most studies focused on self-management as the intended purpose $\left(n=8\right),$ with a minority focusing on well-being with biofeedback $\left(n=2\right)$. Finally, many studies offer passive sensing with no complementary interventions $\left(n=10\right)$.

#### Methodological Quality

From the included studies, that most commonly report study type was that of pilot study (n=4), followed by randomized control $\left(n=3\right)$ and finally cross-sectional study $\left(n=1\right)$. The remaining studies did not report a study type within their methodology $\left(n=3\right)$. A timeframe of 4 weeks was the most common reported $\left(n=4\right)$, followed by 8 weeks $\left(n=2\right)$ and finally 10, 1 and 2 weeks $\left(n=1\right)$, respectively. Most studies focused on the identification of stress response $\left(n=9\right),$ with few targeting stress reduction $\left(n=2\right)$. Additional details such as sample size, target population and target intervention are available in Table 2, and are additionally available within the Supplementary Data—methodological quality.

Table 2 illustrates the study characteristics of those included in this review. From the included references, sample size, demographic indicators, targeted mental health disorder and intended purpose are included. Additionally, Boolean values for passive sensing and intervention indicate whether the included study supports them. Finally, the individual cell is left blank where no details are available. * Unless stipulated within the included study, the age range is derived from the given target group per [26].

**Table 2:** Study characteristics including demographic and target mental health disorder.

Study		Demographic					Mental Health Disorder			
Reference	Sample Size	Participants (Male %)	Participants (Female %)	Participants Age Range	Participant’s Age (Mean)	Target Group	Targeted Disorder	Intended Purpose	Passive Sensing	Intervention
[27]	55	0.69	0.31	18–25	23.2	Young Adults	Anxiety	Well-being (Biofeedback)	N	Y
[28]	55	0.88	0.12	33–59	46.5	Adults	Depression	Detection	Y	N
[29]	183			33–59		Adults	Stress	Self-Monitoring	Y	N
[30]	23	0.7	0.3	22–56	30.35	Employees	Stress	Validation	Y	N
[31]	20			33–59		University Students	Depression	Self-Monitoring	Y	N
[32]	201	0.55	0.45	18–25		University Students	Stress, Depression, Anxiety	Self-Monitoring	Y	N
[33]	82	0.35	0.65	17–38		University Students	Stress	Self-Monitoring	Y	N
[34]	169	0.45	0.55	33–59	33	Employees	Stress	Well-being (Biofeedback)	Y	Y
[35]	328	0.57	0.43	33–59	38.9	Employees	Stress	Self-Monitoring	Y	N
[36]	5895						Depression	Self-Monitoring	Y	N
[37]	80	0.50	0.50	50–70		Older Adults	Depression	Self-Monitoring	N	N
[38]	196	0.33	0.77	28.8–48.4		Employees	Anxiety	Self-Monitoring	Y	N

### 5.2. Wearable Devices and Device Types

From the perspective of wearable device type, perhaps unsurprisingly, ‘smartwatches’ are the most targeted device within the included technologies $\left(n=6\right),$ with the highest distribution between 2019 and 2020. The other device types included headbands $\left(n=2\right)$, glasses $\left(n=1\right)$, wristbands $\left(n=2\right)$, necklaces $\left(n=1\right)$ and shirts $\left(n=1\right)$. Of the devices which included machine learning (ML) components $\left(n=10\right)$, the most common ML model was state vector machines with radial basis function (SVM RBF n=3). Although a typical inclusion in early multi-model testing, the SVM RBF algorithm has been demonstrated to outperform other classification methods for identifying mental states [32,36]. As per the reported accuracy, SVM-based models performed better than other models, M=0.89, min=0.81,  max=0.96 $\left(W=0.985,\;\;P=0.931\right)$ compared to other methods, including neural networks, statistical mixed design models, random forest and binary logistic regression models M=0.83,  min=0.81,  max=0.96 $\left(W=0.914,\;\;P=0.465\right)$.

The most common self-reporting scale used in conjunction with wearable data was the Perceived Stress Scale (PSS, n=4), followed by the State-Trait Anxiety Inventory (STAI, n=3) and the Hamilton depression scale (HAM-D, n=2). The remaining measurement and evaluation scales are presented in Table 3.

Table 3 illustrates the measurement and evaluation scales used within the included studies. In addition, the respective abbreviation, full-scale name, and record count across the dataset are presented.

Wearable devices and associated algorithms with the other variables extracted and included within the analysis are included in Table 4 below.

Table 4 illustrates the wearable device characteristics, including manufacturer, model, and device type. Additionally, algorithm details such as ML model(s), accuracy, biometric signal, self-reporting scales and evaluation scales for the included studies are also provided. Finally, the individual cell is left blank where no details are available.

### 5.3. Wearable Device Data Type

As per Table 5, 13 metrics were used across the dataset. First, the most referenced metrics (n=9) were Electrodermal activity (EDA/GSR/SC/Skin Temperature), which are indicators of emotional arousal via sweat gland activity [39,40,41,42]. Next, heart rate (HR) (n=6) and physical activity (n=6). Finally, the least common biometrics were Electrocardiogram (ECG), Electroencephalography (EEG), Respiratory Rate and Calorie intake with two, two, two, and one references, respectively.

Table 5 presents the biometrics used within the wearables included in this review and their respective instance count. In addition, the collection method, wearable location and metric description are also given.

Physical activity is typically an aggregate of step count, motion, accelerometer, or gyroscopic data and is a standard metric, particularly in smartwatches [23]. There is a significant body of literature supporting the positive relationship between physical activity and mental health, with recent literature exploring this relationship considering the recent COVID-19 pandemic [54]. Marvaldi et al [7], found that higher levels of physical activity were significantly associated with lower levels of mood disturbance and less states of anxiety, with days of physical activity per week being a strong predictor of mental state.

However, it suggested that physical activity is not necessarily proportional to its inverse of sedentary behaviour, where the imposition of such conduct harms mental states [54]. Additionally, the extent to which physical activity may mitigate the effects of sedentary behaviour is unknown [54]. Similarly, the metrics used to determine physical activity are regularly reported as being a point of fixation among wearable users, where they may ignore other evidence of health issues [2].

### 5.4. Economic Analysis of Technologies

Many mental health technologies rarely report any cost–benefit within their release, focussing rather on short-term gains, a trend equally true for the technologies included in this review [3]. To quantify the economic benefit of technologies, we consider the public value benefit as per the Greater Manchester cost-benefit analysis (CBA) model, which estimates the return on investment (ROI) for public sector interventions [25]. This tool requires making a series of assumptions based on the target population, potential impact, engagement and deadweight (business-as-usual) and, therefore, should be treated as an approximation of the return on investment for such technologies [25,55].

#### 5.4.1. Domains

Economic analysis is based on the ROI across various domains [25,55]. As such, the domains applicable to mental health technologies for self-management of subclinical common mental health disorders are the following domains as per Table 6.

#### 5.4.2. Assumptions

For calculation of the public value benefit per the Greater Manchester cost-benefit analysis (CBA) model, the following assumptions are made: target and affected populations, level of engagement with the target population, retention, impact and deadweight [25]. The following sections detail these assumptions.

**Target and affected population:** The target populations are applied to the UK, per the UK census 2021 initial results, and House of Commons library per target group category and age range [56]. Thus, within the included studies, as of 2020, unless otherwise stipulated, five population groups are defined and quantified as young adults (n=5.2 M, [56]), adults (n=67.1 M, [56]), older adults (n=11.8 M as of 2016, [56]), employees (n=30.3 M, [56]) and university students (n=2.66 M, [57]) are targeted. Similarly, the affected population within these groups are determined by the percentage of people who experience subclinical common mental health symptoms and are therefore likely to find the technology of relevance. Thus, the targeted population for common mental health disorders per each group are young adults (n=1.8 M, [56,58]), adults (n=13.8 M, [56,59,60]), older adults (n=3 M, [56,61,62]), university students (n=0.8 M, [57,63]), and employees (n=7.7 M, [56,59,60]). Full details of the targeted and affected populations are given in Supplementary Data—economic and population data.

**Level of engagement with target population:** is based upon the technology type ownership and therefore acts as a ceiling estimate of potential engagement. Technologies were grouped by type, and then the estimated UK ownership of each technology type was explored and expressed as a percentage. Perhaps unsurprisingly, fitness trackers in the form of bracelets or wristbands had the greatest ownership among technology types, estimated at 0.14 [53]. Smartwatches followed this at 0.13 [64,65]. Smart headbands, glasses, and clothing had the lowest penetration at <0.05; therefore, the default upper bound estimate of 0.05 was taken for completeness. Full details for the level of engagement per technology type are given in Supplementary Data—economic costs data, level of engagement.

**Retention:** The attrition rate attributed to mental health apps and technologies is widely documented and commonly regarded as a significant challenge affecting mental health technology adoption, particularly for self-management of common mental health disorders [11]. Similar to engagement, the retention rate of various technologies is estimated based on the technology type and expressed as retained percentage of population after 6 months. For example, the highest retention rate was attributed to fitness trackers such as wristbands or bracelets, 0.58–0.74 with an average of 0.66 [53,66], followed by smartwatches at 0.23–0.63 with an average of 0.43 [64,65].

**Impact:** There is significant variation in reports for the level of individual behaviour augmentation with wearable devices, particularly concerning common mental health disorders [67]. However, the most significant impact comes from behaviour modulation concerning physical activity (0.43–0.51 [68]). Therefore, for this estimation, we used the impact suggested by Gal et al [68] at 0.43 as a proxy for mental health augmentation impact.

**Deadweight:** is a common statistic cited within the social return on investment estimations as the general rate at which an individual’s well-being will improve regardless of intervention [55]. Banke-Thomas et al [69] cite the deadweight range for public bodies between 0.18 and 0.43. Thus, we take the mid value of 0.33 as an approximation of deadweight.

#### 5.4.3. Public Value Benefit

**Figure 3:**
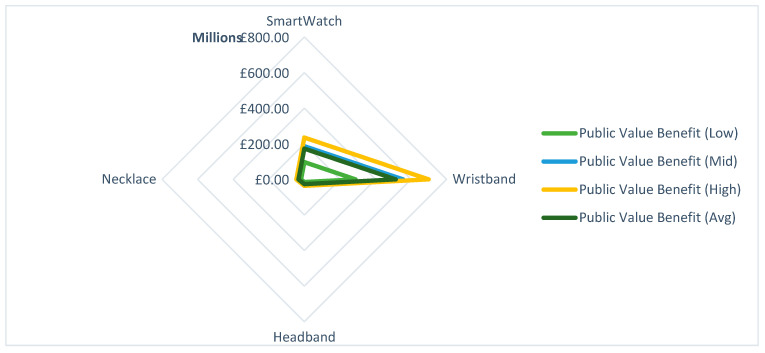
Public value benefit for wearable technologies included within this review.

Of the technologies included within this review, wristbands, including fitness trackers and bracelets, had the greatest public value benefit estimate, at an average of £513.9 M. Surprisingly, smartwatches resulted in similar public value benefits to necklaces, £172.9 M. This is perhaps contrasting as, despite the high penetration of smartwatches compared to other wearable technologies, their public value benefit is 0.33 of fitness trackers despite greater penetration and retention. This is due to many studies focusing on the application of smartwatches to specific domains $\left(n=7\right)$ compared to fitness trackers $\left(n=2\right)$ and necklaces $\left(n=1\right)$. These particular domains, such as university students or employees, have reduced population and target population compared to the broader adult population-focused technologies. Thus, results are likely skewed in this respect. Headbands had the least public value benefit at £26.7 M. This is further illustrated in Figure 3.

## 6. Discussion

We sought to evaluate the utility of wearables for the self-management of sub clinical, common mental health disorders. In doing so, the following critical perspectives were explored (1) the dominant wearable biometrics, (2) dominant purpose of wearable devices, (3) the dominant machine learning algorithms at work within these devices, and finally (4) the potential cost–benefit of such devices.

Of the 12 wearable devices included in this study, electrodermal activity (EDA/GSR/SC/skin temperature), physical activity, and heart rate (HR) are the most common biometrics with nine, six and six reference counts, respectively. Self-management of stress or anxiety where the most common application (*n* = 8) with smartwatches being the most common targeted device (*n* = 6). State Vector machine with radial basis function (SVM-RBF) algorithms comprise the dominant machine learning component where classification of mental states is included within the device (*n* = 3), SVM RBF algorithm has been demonstrated to outperform other classification methods for identifying mental states [32,36]. Finally, fitness trackers and bracelets had the greatest public value benefit estimate, at an average of £513.9 M while the lowest belonged to headbands at £26.7 M.

Among the barriers to integration, wearables offer three principal challenges and opportunities: (1) they have the potential to holistically empower the therapeutic process, giving individuals a greater degree of control and input to their treatment while giving them the necessary tools to implement self-management and preventative strategies at home; (2) There is a significant lack of data available from wearable device manufacturers which validates the assumptions or claims that such devices are beneficial to users’ mental health; Finally (3) wearable devices are significantly more likely to be purchased by those already conscious of their health and therefore greater effort is needed to engage those outside of this scope.

### 6.1. Opportunities

There are three principal opportunities identified throughout this review: (1) Wearables as a ubiquitous delivery method and longitudinal data collection mechanism; (2) Widening access of wearables; and finally (3) Mental health technologies as a means to empower the therapeutic process.

#### 6.1.1. Wearables as a Data Collection Platform

Smart watches, and bracelets such as Fitbit have the potential to become a biometric platform for improving physical performance and positive habit formation, giving individuals direct access to personal analytics, which can assist self-management of health and facilitate preventative care.

Piwek et al. 2016, suggest that wearable devices could provide detailed and cost-effective longitudinal data without the need to involve more sophisticated, uncomfortable, and expensive alternatives. Furthermore, Piwek et al [23], suggests that there is a growing body of evidence to suggest that through data collected by wearable devices, it is possible to determine the severity of depressive symptoms using metrics such as physical activity, sleep duration, and self-reported number of conversations. This view is supported by Kang and Chai [42], who suggest that the increased and changing needs of mental health services have elevated the potential role of sensors for monitoring mental status, and providing data which would be timely and accessible to a range of professionals and users.

Sadeh-Sharvit and Hollon [70] argue that technology has the potential to bring about measurement-based care (MBC), defining measurement-based care (MBC) as the ‘practice of grounding clinical care in patient data collected throughout treatment’. Service providers who utilise such techniques will help individuals achieve faster and more significant treatment responses with symptomatic remission [70].

#### 6.1.2. Widening Access to Treatments through Wearables

In 2016 as part of England’s NHS ‘Improving Access to Psychological Therapies (IAPT)’ program, waiting list standards for psychological treatment were introduced [6]. The waiting list standard states two targets (1) that 75% of referrals for psychological treatment will be assessed and actioned within six weeks, and 98% within eighteen weeks [6]. Despite such targets being reportedly marked achieved, the outcomes across the UK are not uniform, with some areas waiting up to twenty-three weeks for an initial consultation or follow-up session [6]. Thus, despite considerable increases in treatment resources, the service provision gap remains prominent [71].

It is well established that early intervention is effective at limiting the development of common mental health disorders, comparable to that of community mental health care [3,72]. However, considerable challenges in mental health services result in such early interventions often being neglected, particularly in the case of sub clinical mental health symptoms [11,19]. 

The estimated ownership of each technology type per the previous CBA analysis suggests that wearable technologies do indeed represent a high sizeable market penetration at 0.12 of UK adults. According to OFCOM [73] 0.85 of UK adults use a smartphone. The highest ownership belonging to adults aged 16–34 at 0.96 and lowest to adults aged 65+ years at 0.55.

Therefore, the use of digital devices to access mental health services or manage mental health symptoms, particularly for those already engaged with technology, is becoming ever more significant [12]. Digital devices can promote improved access to services for those from low socioeconomic groups or those who live in rural areas while promoting empowerment and participation [12].

#### 6.1.3. Empowering the Therapeutic Process

Sadeh-Sharvit and Hollon [70], suggest that psychotherapy is currently on the edge of a technological transformation. Furthermore, Sadeh-Sharvit and Hollon [70] illustrates that while psychological treatments for common mental health disorders have demonstrated efficiency, their overall quality inclusive of barriers such as access to treatment, cost, lack of objective and systematic methods for assessing treatments during delivery, is poor. However, there is growing evidence that from the perspective of health professionals, wearables have the potential to mitigate against these barriers, while empowering both professionals and end users to (a) reduce subjective influence during diagnosis, (b) develop more individual or bespoke treatment options, and (c) reduce burden on services through appropriate triage of individuals [11,23,70].

Much of the criticism around mental health technologies, particularly those used in conjunction with, or during therapeutic intervention is that they may introduce barriers such as time to administer, collect and analyse data, and therefore may interfere with rapport and the therapeutic alliance [70]. Sadeh-Sharvit and Hollon [70] suggests that mental health tools, to be maximally effective, must collect, segment, and analyse data passively without increasing the therapist burden or reducing face-to-face communications.

### 6.2. Limitations

Three principal limitations were identified throughout this review (1) lack of economic data to support the true cost-benefit of wearables, (2) lack of empirical evidence to support the efficacy of wearables and (3) poor penetration of wearables which results in the potential of such technologies remaining with those already technologically engaged, thus exacerbating the digital divide.

#### 6.2.1. The True Economic Case for Wearables

Economics and mental health have a complex bi-directional relationship. For instance, economic disadvantage is associated with a greater incidence of mental health through exposure to greater risk factors, the latter compounding the former and vice versa [3,5].

According to Knapp & Wong [5], three principal economic evaluations are used to assess mental health interventions. These are (1) cost-effectiveness analysis (CEA), (2) Cost-Utility analysis (CIA) and (3) Cost-Benefit Analysis. Unlike CEA and CIA analysis techniques, CBA places a monetary value on multiple outcomes expressed in net benefits [5]. Such benefits are defined as monetary values of effects minus cost changes [5].

As the economic burden of mental health continues to rise, innovative technologies, processes and services are required to widen access and efficiently manage and treat mental health disorders [3,11]. Digital therapies and wearable devices have great potential to empower users and enhance existing services [11]. However, associated savings with such technology may be offset by their reduced effectiveness [5].

Such complexity, unfortunately, results in the economic analysis for new technologies being avoided despite an imperative need for the complete assessment of new technologies [74].

#### 6.2.2. Lack of Evidence Base

Despite the clear potential of wearables, their implementation remains hampered by attrition either due to lack of motivation on behalf of the user, or poor trigger, engagement, or even identification of needs on behalf of developers and most importantly, lack of supporting evidence [11,23,39,75]. Additionally, although many wearable devices act as a platform for data collection with many biometrics having their own supporting evidence, the efficacy of the platform at large and its application to mental health is unsubstantiated [23].

Much of the evidence provided in support of wearables or digital mental tools remains anecdotal, with few manufacturers or developers providing supporting empirical evidence [11,75]. Piwek et al [23] argue that manufacturers of wearable devices or those who market them, potentially underestimate the distance between designing a product to support a healthy lifestyle and providing the evidence necessary to support this assumption. Similarly, most available mental health applications are not based upon behaviour change theory, and thus any benefit is likely short-lived [13,14]. Huckvale et al [13], during their evaluation of mental health apps, showed that no statistical difference arose from mental health apps with digital control that contained no therapeutic content, thus raising the prospect of digital placebo effects. This supports the findings of Firth et al [11] who argue that the resultant psychological improvements are likely due to an individual’s personal connection with their device and frequent engagement with mental health apps while pursuing expected benefits rather than actual efficacy.

Therefore, to realise the potential of mental health technologies as a valid intervention either for the self-management of common mental health disorders or integrated into clinical practice, manufacturers and developers must ensure their technology is (1) developed with the specific needs and perspectives of their target population, and (2) ensure that such technologies are rigorously tested with respect of safety and efficacy [11,14,75].

#### 6.2.3. Empowering the Already Empowered

Despite the apparent potential of wearables Piwek et al [23] suggest that wearables are more likely to be purchased by individuals who have already adopted a healthy lifestyle and seek to qualify their progress. There is also a growing subculture that uses wearables for the purpose of self-discovery through personal analytics, as seen in the quantified self (QS) movement [23]. This aligns with user perceptions toward ‘self-hacking’, that is, the use of wearable devices to improve sleep, manage stress or increase productivity [23].

The use of mental health tools is correlated with their perceived effectiveness and knowledge of how these tools work [71]. In general, 32% of users stop using wearable devices after 6 months, while 50% stop after 1 year, which is typical of attrition of mental health technologies, particularly mental health apps [11,23]. The utilisation of behavioural theories and persuasive design is employed by developers to combat these high attrition rates [11,12].

#### 6.2.4. Limitations of This Review

This review has a number of limitations, the most obvious of which is the small number of included studies $\left(n=12\right)$. Additionally, this review excludes studies which do not include a physical device, therefore omitting the contributions of mobile apps and such companion technologies. Finally, this review excludes studies which do not focus on common mental disorders as their primary target, therefore including studies which apply wearable devices to physical disorders such as diabetes where symptomatic reduction in mental health disorders using wearables is secondary to the management of the participants physical disorder.

## 7. Conclusions

Despite compelling evidence of intervention that reduces the impact of depressive or anxiety disorder, no reduction in their global prevalence or burden has been detected since 1990 [1]. Despite evidence and a seemingly abundance of preventative and intervention tools and techniques, depressive and anxiety disorders remain the leading health burden globally [1,76,77,78].

Whilst a consistent issue, individuals have become less likely to seek care for mental health problems compared to pre-COVID-19 due to concerns they may become infected as a result [1,79,80,81]. Therefore, a need exists to integrate mental health responses within the COVID-19 recovery strategy [1]. It is argued that recovery strategies should promote mental well-being and specifically target the determinants of poor mental health exacerbated by population shock events such as the COVID-19 pandemic, with intervention being fundamentally important for those who develop such a disorder [1,79,80,81].

This review has evaluated the potential utility of wearables for the self-management of sub-clinical common mental health disorders, the outcome of which has provided more understanding of the potential of wearables to reduce the burden on the healthcare system. Mental health technologies require greater evidence from case reports, randomised control trials and meta-analyses to render such technologies pertinent, and thus further empirical support for their application in the clinical setting is required [70].

## Figures and Tables

**Table 1:** Details the initial search terms, the quantity of returned results and the originating database.

Term	Google Scholar	Web of Science	Scopus	PubMed	Cochrane Library	PsycINFO	Total
Review AND (Anxiety OR depression)	3,110,000	105,939	156,452	95,590	6716	52,223	3,526,920
(“sleep disorders” OR (Common mental health disorders))	3,150,000	60,990	104,776	50,117	10,706	46,173	3,422,762
(“self-management” OR “self-care”)	17,900	52,266	89,471	64,278	0	30,781	254,696
(“subclinical” OR “home management”)	18,100	4661	5402	3858	599	1095	33,715
(Wearables OR “wearable devices” OR “Smart Devices” OR “measurement device” OR “monitoring device” OR “smart wearables”)	157,000	24,508	115,854	25,605	3469	1225	327,661
(Remote OR sensor OR “sensing device”)	7,160,000	1,509,175	1,877,800	301,094	12,259	19,868	10,880,196

The complete search term is: ‘Review AND (Anxiety* OR depress*) AND (“sleep disorders” OR (Common mental health disorders)) AND (“subclinical” OR “home management”) AND (“self-management” OR “self-care”) AND (remote OR sensor OR “sensing*”) AND (Wearables OR “wearable devices” OR “Smart*” OR “measurement device” OR “monitoring device”)’.

**Table 3:** Evaluation scales including abbreviation, full name and record count within the included studies.

Abbreviation	Measurement Scale Full Name	Record Count
HAM-D	Hamilton depression rating scale	2
BDI (BDI-II)	Beck Depression Inventory (I, II)	1
BSI	Brief Symptom Inventory	1
STAI	State-Trait Anxiety Inventory	3
PSS	Perceived Stress Scale—PSS	4
POMS	Profile of mood states	1
PHQ-9	Patient Health Questionnaire-9	1
EMA	Ecological Momentary Assessment	1
MASQ	Mood and Anxiety Symptoms Questionnaire	1
MADRS	Montgomery–Åsberg Depression Rating Scale	1
SRI	Stress Response Inventory	1
CDC HRQOL-14	Centre for Disease Control’s Healthy Days Core and Symptoms Modules	1

**Table 4:** Wearable device characteristics for their associated study.

Wearable Device			Algorithm					
Manufacturer	Wearable Model	Device Type	ML Model(s)	Accuracy	Biometric Signals	Self-Reporting Scales	Evaluation Scales	Reference
InteraXon Inc & SmithOptics Inc.	Muse™, Lowdown Focus	Headband, Glasses		0.77	EEG, HRV	PSS, POMS, STAI	BDI, BSI	[27]
Eee Holter Technology Co.	Mindo-4S Jellyfish	Headband	SVM RBF	0.81	EEG	HAM-D		[28]
Empatica	E4	Wristband	Binary logistic regression model	0.85	GSR			[29]
Apple	Watch 6	SmartWatch			HR			[30]
Samsung	Gear S3 Frontier	SmartWatch	SVM RF	0.96	Physical Activity, HR	EMA, PHQ-9	BDI-II, STAI	[31]
Affectiva	Q-sensor	SmartWatch	SVM RBF	0.87	Physical Activity, SC, S-Temp, Ambient light	PSS		[32]
Microsoft	Smartband 2	SmartWatch	Neural Network (NN)	0.78	SC, Sleep, Calorie intake, S-Temp, HR, HRV, PPG, RR	STAI	PSS, SRI	[33]
Spire Health	Spire Stone	Necklace	-	-	Respiratory Rate	MASQ	CDC HRQOL-14	[34]
Intelligent Galaxy	Chillband	SmartWatch	Statistical mixed design model	0.75	Physical Activity, ECG, SC, S-Temp, HR, Circadian Harmonic	PSS		[35]
Cambridge Neurotechnology Ltd.	Actiwatch	SmartWatch	Feature extraction and RF	0.89	Physical Activity	MADRS		[36]
Acculi Labs Pvt. Ltd.	LENS	Bracelet	-	0.93	Physical Activity, S-Temp, menstrual cycle, Sp02, Sleep monitoring, PPG	HAM-D		[37]
FitBit, OMsignal	OMsignal smart-shirt, Fitbit Charge 2	SmartWatch, Smart Shirt	SVM RBF	0.92	Physical Activity, HR, HRV, Sleep, ECG, PPG, RR, GSR	daily survey		[38]

**Table 5:** Biometrics reference count and description.

Biometric	Collection	Count	Location	Description	Reference
Physical activity	Auto/Self-reported	6	On person	Physical activity is a commonly used metric default in many consumer wearable devices, which includes acceleration and step counts.	[2,14,43]
Electrodermal activity (EDA/GSR/SC/Skin Temperature)	Auto	9	Wrist	Electrodermal activity (EDA), galvanic skin response (GSR) and skin temperature are measures of skin conductance indicative of sweat gland activity and, therefore, emotional arousal.	[39,40,41,42]
Blood oxygen saturation (Sp02)	Auto	1	Wrist	Blood oxygen saturation (SP02) is a common biometric within clinical practice and is typically collected via pulse oximetry, reflecting the percentage of oxygen in the blood.	[44,45,46,47]
Heart Rate (HR)	Auto	6	Wrist	Heart Rate is the number of heart beats per minute	[41,47]
Heart Rate Variability (HRV)	Auto	3	Wrist	Heart rate variability (HRV) is a variation of the interval between heartbeats.	[41,47]
Sleep	Auto/Self-reported	3	Wrist	Changes in sleep patterns are commonly associated with signs of mental health deterioration. Therefore, sleep patterns are regular indicators of mental health status.	[43,48,49]
Ambient light and audio	Auto	1	Wrist	Ambient light or audio is a commonly used metric in conjunction with physical activity and SC to determine sleep activity and quality.	[31,43]
Menstrual cycle	Self-reported	1	Off person	Psychological stress has a detrimental effect on menstrual cycle regularity.	[50]
Photoplethysmography (PPG)	Auto	3	Wrist	Commonly used metric to determine the amount of light absorbed by blood vessels in living tissue. PPG can be used as a proxy for blood pressure due to the correlation between arterial blood volume and distention with blood pressure.	[47,51,52]
Electrocardiogram (ECG)	Auto	2	Wrist	A plot of the heart’s electrical activity is traditionally used to calculate HR and HRV	[46]
Electroencephalography (EEG)	Auto	2	Head	Electroencephalography (EEG) is a measure of the brains electrical activity over time and is one of the most effective physiological signals for identification of psychological stress.	[41,46]
Respiratory Rate	Auto	2	Chest	Respiratory patterns, i.e., inspiration/expiration ratio, respiratory pauses, irregularity etc. are influenced by various mental stressors and therefore is a common indicator of mental state.	[42]
Calorie intake	Self-reported	1	Off person	Calorie intake indicators are typically self-reported and are of interest because food intake is shown to be correlated with depressive symptoms	[31,53]

**Table 6:** Domains of target outcome, benefit and recipient as per 25.

Outcome	Benefit	Recipient
Improved well-being of individuals	Increased confidence/self-esteem	Individual
	Reduced isolation	Individual
	Positive functioning (autonomy, control, aspirations)	Individual
	Emotional well-being	Individual
Improved family well-being	Improved family relationships	Family
	Positive functioning (autonomy, control, aspirations)	Family
	Emotional well-being	Family
Improved community well-being	Sense of trust and belonging	Community
	Positive functioning (autonomy, control, aspirations)	Community
	Improved relationships	Community
Mental health	Reduced health cost of interventions	NHS/Individuals

## Data Availability

Technologies and economic data is provided within the two enclosed Supplementary Files. Technologies includes all metrics collected from the included studies while economic data includes level of engagement and population data.

## Supplementary Materials

The following supporting information can be downloaded at: https://www.mdpi.com/article/10.3390/ijerph20032636/s1.
